# Effect of Low Temperature Cultivation on the Phytochemical Profile and Bioactivity of Arctic Plants: A Case of *Dracocephalum palmatum*

**DOI:** 10.3390/ijms18122579

**Published:** 2017-11-30

**Authors:** Daniil N. Olennikov, Nadezhda K. Chirikova, Nina I. Kashchenko, Tat’yana G. Gornostai, Inessa Yu. Selyutina, Ifrat N. Zilfikarov

**Affiliations:** 1 Institute of General and Experimental Biology, Siberian Division, Russian Academy of Science, Sakh’yanovoy Street 6, 670047 Ulan-Ude, Russia; ninkk@mail.ru; 2 Department of Biochemistry and Biotechnology, North-Eastern Federal University, 58 Belinsky Street, 677027 Yakutsk, Russia; hofnung@mail.ru; 3 Siberian Institute of Plant Physiology and Biochemistry, Siberian Division, Russian Academy of Science, Lermontova Street 132, 664033 Irkutsk, Russia; t.g.gornostay@yandex.ru; 4 Central Siberian Botanical Garden, Siberian Division, Russian Academy of Science, Zolotodolinskaya Street 1, 630090 Novosibirsk, Russia; selyutina.inessa@mail.ru; 5 All-Russian Institute of Medical and Aromatic Plants, Greena Street 7/1, 117216 Moscow, Russia; zilfikarovin@mail.ru

**Keywords:** *Dracocephalum palmatum*, fatty acids, essential oil, phenolic compounds, carbohydrates, low-temperature cultivation, antioxidant activity, gas chromatography-mass spectrometry (GC-MS), high performance liquid chromatography with diode array detection and electrospray ionization mass-spectrometric detection (HPLC-DAD-ESI-MS)

## Abstract

The influence of climatic factors, e.g., low temperature, on the phytochemical composition and bioactivity of the arctic plant *Dracocephalum palmatum* Steph. ax Willd. (palmate dragonhead), a traditional food and medical herb of Northern Siberia, was investigated. *D. palmatum* seedlings were grown in a greenhouse experiment at normal (20 °C, NT) and low (1 °C, LT) temperature levels and five groups of components that were lipophilic and hydrophilic in nature were characterized. The analyses indicated that *D. palmatum* under NT demonstrates high content of photosynthetic pigments, specific fatty acid (FA) profile with domination of saturated FA (53.3%) and the essential oil with trans-pinocamphone as a main component (37.9%). Phenolic compounds were identified using a combination of high performance liquid chromatography with diode array detection and electrospray ionization mass-spectrometric detection (HPLC-DAD-ESI-MS) techniques, as well as free carbohydrates and water soluble polysaccharides. For the first time, it was established that the cold acclimation of *D. palmatum* seedlings resulted in various changes in physiological and biochemical parameters such as membrane permeability, photosynthetic potential, membrane fluidity, leaf surface secretory function, reactive oxygen species–antioxidant balance, osmoregulator content and cell wall polymers. In brief, results showed that the adaptive strategy of *D. palmatum* under LT was realized on the accumulation of membrane or surface components with more fluid properties (unsaturated FA and essential oils), antioxidants (phenolic compounds and enzymes), osmoprotectants (free sugars) and cell wall components (polysaccharides). In addition, the occurrence of unusual flavonoids including two new isomeric malonyl esters of eriodictyol-7-*O*-glucoside was found in LT samples. Data thus obtained allow improving our understanding of ecophysiological mechanisms of cold adaptation of arctic plants.

## 1. Introduction

Environmental stress is one of the most important factors limiting the productivity of the majority of economically important crops [1]. Extreme growth conditions can damage the plant habitat, their inner ultrastructure and biochemical composition, causing dramatic changes incompatible with the functions of living organisms [2]. To elevate their vitality, plants have developed the remarkable ability to adapt to severe environmental conditions. These specific potentials have allowed the use of plants as crops even in areas with extremely uncomfortable climate conditions [3].

The territories of the Arctic floristic region of Siberia and the Republic of Sakha (Yakutia) belong to an area of high-risk farming due to unfavorable environmental conditions complicating the implementation of agronomic measures. In the period of maximum growth (vegetation) of agricultural plants (May–September), short-term drops of night temperatures are possible from +2 (July) to −10 °C (May and September) [4]. To understand agronomical events in regions with similar weather conditions, it is necessary to use specific cultures that are particularly resistant to the effects of extreme abiotic factors, including temperature. *Dracocephalum palmatum* Steph. ex Willd. (syn. *D. schelechowii* Turcz. ex Ledeb., *Ruyschiana palmata* (Steph. ex Willd.) House, palmate dragonhead) is an example of a widespread species in the Lamiaceae family in the northern part of Siberia (Aldansky, Indigirsky and Kolymsky floral regions) in the permafrost territory and within the Arctic Circle in the range of the Arctic floristic region with a high ecological adaptability [5]. Increased resistance to the effects of chilling temperatures and short-term frosts characterizes this plant species, and it can therefore be defined as a cold-tolerant plant [6].

The herb *D. palmatum* has economic value as a medicinal plant and in the food industry as a spice and tea component; therefore, *D. palmatum* is cultivated in the territory of the modern Republic of Sakha (Yakutia) [7]. Early investigations have shown that *D. palmatum* is characterized by the presence of various groups of natural compounds, including phenylpropanoids, flavonoids, coumarins and triterpenes [8,9]. The pronounced antioxidant activity of *D. palmatum* extracts due to the presence of flavone glycosides of luteolin and apigenin explains its application in medical practice as a hepatoprotective remedy [8]. It should be noted that the use of plant species of the genus *Dracocephalum* in human households is common occurrence. *D. moldavica* L. is the most famous *Dracocephalum* species, which is the object of large-tonnage production as an essential oil source and medicinal plant [10]. Moreover, the traditional medicinal plants *D. heterophyllum* Benth and *D. tanguticum* Maxim. are widely used in the medical systems of China and Tibet for the treatment of asthma, bronchitis, gastropathies and hepatitis [11]. In this regard, the application of the local plant species *D. palmatum* as a food and medicinal preparation in the territory of the north of Siberia is quite justified.

Previous research studies have shown the influence of different types of abiotic stress factors on the essential oil content of *Dracocephalum* plants. Alaei et al. reported that high levels of salinity may have resulted in a two-fold increase in the amount of essential oil in *D. moldavica* herb [12]. Studies have also demonstrated that stress levels due to rising water resulted in the decrease of essential oil amounts [13]. It is generally accepted that essential oils are the principal components of *Dracocephalum* plants, similar to *D. heterophyllum* [14], *D. moldavica* [15], *D. kotchii* [16] and others [11]. However, it should not be forgotten that there are many important natural components that are lipophilic (fatty acids and terpenoids) and hydrophilic in nature (phenolics and carbohydrates) that were identified in the *Dracocephalum* genus [11]. There is a complete lack of scientific information about the effect of cold stress on chemical components of non-“essential oil”-nature in *Dracocephalum* species, making it impossible to understand the fundaments of its adaptive process. Possession of the composite data of both lipophilic and hydrophilic compounds variation in *Dracocephalum* species will be useful for creation of an effective strategy for maximizing the production of secondary metabolites.

The present study was designed to understand the physiological mechanism of cold tolerance of *D. palmatum*. The chemical responses of *D. palmatum* seedlings were estimated by analyzing the phytochemical profile of plants cultivated under low (LT) and normal temperature (NT). The following lipophilic groups of compounds were chosen as markers of lipidome homeostasis: chlorophylls and carotenoids due to their role as important and critical biomolecules in photosynthesis with function of light absorbance and light energy transformation; fatty acids as components responsible for the liquid properties of plant cell membranes; and essential oils as a group of structurally variable components with functions closely related to leaf ontogeny and possible role as plant growth regulators. Due to the strong relationship between cold stress level and production of reactive oxygen level in plant cells, the composition of antioxidative components as phenolics was also analyzed. Upon further investigation, particular attention was paid to the profile of the osmoprotectant components (free sugars) and cell wall polymers (polysaccharides). Some physiological parameters such as electrolyte leakage, malondialdehyde concentration and activity levels of superoxide dismutase and catalase in plant tissue were also determined. This research allows improving the theoretical knowledge in the field of ecophysiological adaptation of *D. palmatum* and helps to estimate the usefulness of cold-temperature cultivation of the arctic plants.

## 2. Results and Discussion

### 2.1. Phenotypic Changes, Electrolyte Leakage, Photosynthetic Pigment Content and Parameters of Photosynthesis of D. palmatum during Low-Temperature (LT) Cultivation

We investigated the phenotypic response of *D. palmatum* to low-temperature (LT) stress. Seedlings were cultivated in growth chambers with photoperiod of 16 h light/8 h dark installed under normal temperature conditions (20 °C; NT) for two months. One group of seedlings remained under normal conditions and another group was transferred and subjected to LT (1 °C) for 20 days. The level of LT exposure was close to cold-down periods affected by *D. palmatum* plants in his natural habitat. The time-frame was chosen to avoid the visible plant damages caused by long-term LT application. To evaluate whether period can be applied successfully, 10–30 days LT impact was used. As a result, it was demonstrated that the duration of LT (1 °C) should not exceed 20 days.

After the 20-day period, the seedlings grown in 20 °C were at a height where the plant stems reach an average of 100 mm. During the cold treatment at 1 °C, the seedlings were stockier, with the height of the plant stems reaching an average of 80 mm. A significant increase in the yield of fresh herb was not observed, but leaves of the LT seedlings were a saturated green color and the areas of young leaves were not different from the leaves of the group with moderate temperature conditions.

To evaluate the extent of cell damage caused by cold stress in LT seedlings, electrolyte leakage was measured (Table 1). The *D. palmatum* plants after 20 days of LT exposure presented 1.44-fold higher electrolyte leakage than normal temperature plants, which suggests that the membrane is likely to be impaired in these seedlings subjected to cold stress [2].

The photosynthetic pigments from the leaves of the three groups were measured (chlorophylls, pheophytins, and carotenoids). Total chlorophylls and carotenoid concentration of the LT group was significantly higher (547.40 and 53.31 µg/g FW, respectively) than in NT group (349.30 and 36.86 µg/g FW, respectively), indicating that the photosynthetic pigment levels might contribute to the difference in the photosynthetic capacity among the temperature conditions [17,18]. In contrast, total content of pheophytins in LT plants remained almost unchanged. The extent of pheophytin accumulation under LT stress can play a key role as a measure of chlorophyll damage, which, in this case, is not observed. The ratio of chlorophyll a and b (Chl_a_/Chl_b_) in NT group was 3.61 and reduced by 20.2% under LT conditions. By contrast, the ratio of total chlorophyll content ant total carotene content (ΣChl/Car) in *D. palmatum* plants under LT stress was 10.27, which was 8.3% higher than in NT plants. These changes showed that the LT treatment caused the reorientation of chlorophyll synthesis also with respect to total carotene level. It is also an indicator of a shift in content of light-harvesting complex 2 of a leaf photosystem caused by the impact of the stress factors [19].

The value of carbon assimilation rate in LT plants was lower (5.8 µM CO_2_/m^2^·s) than in plant cultivated at normal temperature (8.3 µM CO_2_/m^2^·s). In addition, the effective quantum yield of PSII (F_v_/F_m_) in plants exposed to LT was slightly lower than the value of NT plants, demonstrating inhibition of photosynthetic processes under cold temperature. A possible explanation given by us for the differences of photosynthesis rate and chlorophyll content in LT plants was the ability of *D. palmatum* to accumulate chlorophylls while low rate of its cold-induced degradation.

The known data demonstrate that, at the seedling stage, the impact of chilling stress increases the concentration of the accessory pigments (chlorophyll b and carotenoids) when compared to chlorophyll a, most likely to increase the photon capture [20]. Moreover, in the case of cold-tolerant lines of crops, more chlorophylls accumulate under cold stress than cold sensitive lines of crops [21]. The results showed that *D. palmatum* adapted to the environmental temperature and can be grown and perform better under low-temperature conditions.

### 2.2. Changes of the Fatty Acids of D. palmatum during LT-Cultivation 

The influence of temperature on the composition of fatty acids in *D. palmatum* was investigated. The total lipid fraction yield of *D. palmatum* grown under NT was 1.24% (of dry plant weight; DW) and the fatty acid composition was characterized by high amounts of palmitic acid (27.9%), linolenic acids (14.6%), linoleic acid (14.1%) and oleic acid (11.3%) (Table 2). Concerning the amount of fatty acids in other species of the *Dracocephalum* genus, the fatty acid composition of *D. kotschyi* oil is composed of a high amount of polyunsaturated fatty acids and contains linolenic acid (61.2%) as the predominant fatty acid followed by oleic (18.1%) and linoleic (13.5%) acids [22]. The amount of linolenic acid in *D. moldavica* oil was 59.4% [23]. These results are in accordance with previous data regarding the Lamiaceous plant fatty acid profile where the authors demonstrated the close ratio of the aforementioned acids in herbs [24,25].

The effect of LT environments on *D. palmatum* resulted in the increase of the total lipid fraction yield to 3.22% DW and the decrease in the relative amount of saturated acids compared with normal temperature conditions (specifically, the amounts of tridecylic, palmitic and arachidic acids were reduced 2.8, 2.5 and 9 times, respectively) and an increase in the amount of unsaturated fatty acids (γ-linoleic, gondoic and erucic acids increased 3.5, 1.9 and 1.7 times, respectively). The influence of LT conditions on the *D. palmatum* caused a shift in the saturated/unsaturated ratio compared with cultivation in NT (24.0/75.0 vs. 53.3/46.5). Saturated palmitic acid was the predominant fatty acid in *D. palmatum* cultivated in NT conditions (27.9%), while polyunsaturated linoleic acid dominated in samples that grew under LT conditions (19.3%).

Fatty acids play an important role in the protection of cell membrane against negative effects of long-tern cold exposure [26]. The dominant effect of LT on the lipid composition of cell membranes is the rising level of unsaturated components (e.g., fatty acids) which makes membrane more fluid [27]. This is confirmed by the fact of high content of unsaturated fatty acids (75%) in *D. palmatum* herb cultivated under cold temperature.

### 2.3. Changes of the Essential Oil Profile of D. palmatum during LT-Cultivation

The samples of *D. palmatum* herbal essential oil (EO) were isolated by hydrodistillation and the composition was determined for the first time after gas chromatography-mass spectrometry (GC/MS) analysis. EO are greenish liquids characterized by specific smells caused by the presence of odoriferous components. Thirty-eight compounds were identified in three samples of EO from *D. palmatum* cultivated under different temperatures, including aliphatic compounds, simple phenols, monoterpenes and sesquiterpenes (Table 3).

The yield of the EO was 1.1% for the sample cultivated in moderate environments (20 °C) and 3.7% for *D. palmatum* grown in LT environments. There were no significant differences in the quantitative amounts of aliphatic compounds and simple phenols at both temperatures of cultivation. The predominance of bicyclic monoterpene trans-pinocamphone was noticed at all temperature conditions ranging from 37.9% to 40.7%. The effect of LT environments on *D. palmatum* EOs resulted in a slight increase in the amount of monoterpenes and a significant decrease of total sesquiterpenes compared with growth under normal temperature conditions. Thus, the amount of camphene, *cis*-pinocarveol and *cis*-pinocarvyl acetate increased 2, 2.5 and 1.8 times compared with samples grown at 20 °C. A decrease in the levels of α-tujene, β-myrcene, phellandral and *p*-mentha-1,4-dien-7-ol at 1.5, 3, 2 and 4 times, respectively, was noted.

The total amount of sesquiterpenes decreased 2.9 times compared with samples grown under normal temperature conditions. It can be concluded that temperature environmental conditions should be carefully considered in the cultivation of *D. palmatum* to get the desired concentrations of terpenoids.

The literature regarding the chemical composition of the EO of the *Dracocephalum* genus indicates the similarity of the volatile compounds. Most dominant compounds of the *Dracocephalum* genus related to monoterpenes. *D. moldavica* EO is the most studied. The main components were acyclic monoterpenes *E*-citral (30.4%), geranyl acetate (29.6%) and neral (22.1%) [28]. Other isomers of citral (geranial (63.4%), limonene (23.4%) and *p*-menth-1-en-9-ol (4.4%)) dominated the EOs from the aerial portion of *D. subcapitatum* [29]. The predominance of the acyclic monoterpenes perilla aldehyde and limonene was revealed in the EO from the aerial portions of *D. surmondinum* (54.3% and 30.1%, respectively) [30], *D. multicaule* (71.5% and 28.1%, respectively) [31] and *D. polychaetum* (63.4% and 22.1%, respectively) [32]. Other acyclic monoterpenes such as *p*-mentha-1,8-dien-10-al (39.2%), limonene (17.0%) and geranial (4.6%) prevailed in the EO from the aerial portion of *D. foetidum* [33]. The EO from the aerial portion of *D. heterophyllum* contained citronellol (74.9%), citronellyl formate (6.7%) and citronellal (6.7%) [34]. Volatile constituents of the aerial portion of *D. peregrinum* contained monocyclic monoterpene 1,8-cineol (18.5%), α-pinene (8.4%) and limonene (5.8%) [35]. The predominance of acyclic and bicyclic monoterpenes was noticed in the EO from the aerial portion of *D. kotschyi*, including neral (11%), α-citral (12%) and α-pinene (10%) [36]. *D. aucherry* was a species with a prevalence of EO from the flowering shoots that contained bicyclic monoterpene sabinene (55.2%), germacrone (9.9%) and α-thujene (5.5%) [37]. Another species with a predominance of bicyclic monoterpenes in the EO from aerial portion was *D. wallichii* (*D. speciosum*) (trans-pinocarvyl acetate (60.5%) and *cis*-pinocarvyl acetate (5.7%)) [38].

Temperature levels have been previously reported to influence the EO amount in several aromatic crops of the Lamiaceous group [39]. A decrease in temperature may favor the accumulation of various groups of volatile terpenoids in EO glands on the leaf surface [40]. In sage leaves (*Salvia officinalis* L.), the largest percentage of monoterpenes was observed in the cold period of cultivation (October) [41]. The same effect on monoterpene accumulation was reported for peppermint EO (*Mentha piperita* L.) isolated from the plants grown at LTs [42,43]. The data obtained indicated a similarity of the temperature-induced physiological responses previously detected in Lamiaceous plants and *D. palmatum*. In this way, the production of EO in *D. palmatum* is not only exerted in a development-specific fashion but is also highly susceptible to modulation through temperature regulation.

### 2.4. Changes of Phenolic Compounds of D. palmatum during LT-Cultivation

**Figure 1:**
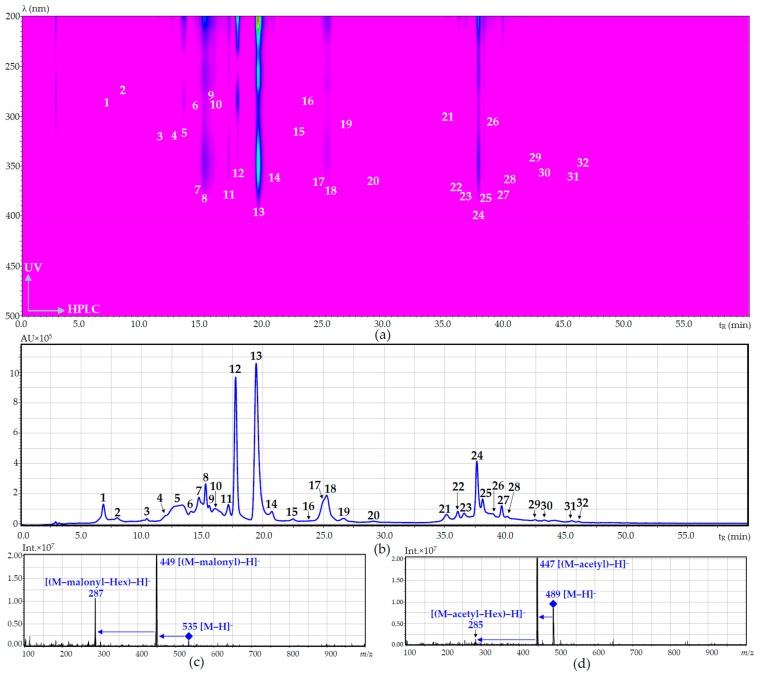
HPLC-UV map (**a**); and RP-HPLC-DAD chromatogram at 280 nm (**b**) of low-temperature sample of *D. palmatum* herb; ESI-MS spectra of unknown compounds **6** (eriodictyol-*O*-malonyl-hexoside) and **20** (luteolin-*O*-acetyl-hexoside) presented at (**c**,**d**), respectively. The numbers in the Figure 1a,b correspond to the compounds indicated in Table 4. AU, absorbance units; Int., signal intensity.

The methanol extracts of *D. palmatum* were analyzed by reversed phase high performance liquid chromatography with diode array detection and electrospray ionization mass-spectrometric detection (RP-HPLC-DAD-ESI-MS) in both negative and positive ionization modes. The HPLC with ultraviolet detection (HPLC-UV) map and HPLC-DAD chromatogram are shown in Figure 1, and chromatographic parameters, UV and ESI-MS data are in Table 4. By comparing the retention times (t_R_), UV and ESI-MS spectra with those of references substances and literature data, 32 components were identified in all plant samples (23 components in NT samples and 32 components in LT sample), including two simple phenolic glycosides (**1** and **2**), four caffeic acid derivatives or phenylpropanoids (**3**–**5** and **19**) and 26 flavonoids (**6**–**18** and **20**–**32**) as glycosides and aglycones.

Two simple phenolic glycosides, compounds **1** and **2**, were detected in *D. palmatum* for the first time. The quasi-molecular ions were [M + H]^+^ as well as sodium adducts [M + Na]^+^ in positive ion mode; both compounds had weak ionization in negative ion mode. Compound **2** was characterized as arbutin (hydroquinone-*O*-glucoside) due to intense [M + Na]^+^ at *m*/*z* 295 and specific absorbance in the UV spectrum compared with the reference compound [44]. Compound **1** gave the sodium adduct [M + Na]^+^ and quasi-molecular ion [M + H]^+^ at *m*/*z* 381 and 352, respectively (i.e., 86 amu more than arbutin indicating malonyl-derivative of **2** tentatively characterized as *O*-malonyl-arbutin) [45]. Data in the literature data demonstrated that the arbutin and its esters are rare components for the Lamiaceae family. Only *Origanum majorana* L. (formerly *Majorana hortensis* Moench.) is known as a good source of arbutin (**2**) as other *Origanum* species (*O. onites* L., *O. microphyllum* (Benth.) Vogel, *O. saccatum* P.H. Davis, *O. solymicum* P.H. Davis) only contain trace amounts of **2** [46]. Arbutin ester **1** was detected only in *D. moldavica* of Mexican origin [45].

Four chromatographic peaks (**3**, **4**, **5** and **19**) with the same UV spectra as the standards of 5-*O*-caffeoylquinic acid, 3-*O*-caffeoylquinic acid, caffeic acid and rosmarinic acid were detected in all samples of *D. palmatum*. Two of them (**3** and **5**) showed the [M − H]^−^ ions at *m*/*z* 353 and one (**19**) at *m*/*z* 359. Components **4**, **5** and **19** were previously detected in *D. palmatum* [8], and **3** was discovered in this species for the first time in this study.

Flavone glycosides as usual components of the flavonoid complex in the *Dracocephalum* genus [11] were presented as derivatives of apigenin, acacetin and luteolin widely distributed in Lamiaceae family [47]. Luteolin-7-*O*-rutinoside (scolymoside, **11**), luteolin-7-*O*-glucoside (cynaroside, **13**), luteolin-4′-*O*-glucoside (**14**), apigenin-7-*O*-rutinoside (isorhoifolin, **15**), apigenin-7-*O*-glucoside (cosmosiin, **17**), acacetin-7-*O*-rutinoside (linarin, **22**) and acacetin-7-*O*-glucoside (tilianin, **23**) standards allowed the unequivocal identification of these seven flavone glycosides in *D. palmatum* extracts. Five of these flavonoids (**11**, **13**–**15**, and **17**) have already been characterized as components of *D. palmatum* [8]. Acacetin derivatives **22** and **23**, described in *D. palmatum* for the first time, are not usual for *Dracocephalum* genus. Both components were also found in *D. foetidum* [48], *D. moldavica* [11] and *D. peregrinum* [49,50].

Two rare di-*O*-glycosides of luteolin, luteolin-7,4′-di-*O*-rutinoside (dracopalmaside, **7**) and luteolin-7-*O*-rutinoside-4′-*O*-glucoside (cynarotriside, **8**), previously isolated from *D. palmatum* of natural origin [9] were also detected in plants grown in greenhouse conditions.

Compound **18** was identified as apigenin-*O*-hexoside according to UV and ESI-MS spectra and literature information [51]. This compound demonstrated the same UV spectra pattern as flavone standards. The ESI-MS deprotonated ion [M − H]^−^ at *m*/*z* 431 and the aglycone fragment at *m*/*z* 269 revealed the characteristic fragmentation of an apigenin-hexose derivative [53]. Due to the difference in the retention times of **18** (t_R_ 25.31 min) and apigenin-7-*O*-glucoside (t_R_ 24.47 min), component **18** may be tentatively determined as apigenin-4′-*O*-glucoside, previously identified in *Elsholtzia rugulosa* Hemsl [51].

Two components, **20** (t_R_ 29.11 min) and **25** (t_R_ 38.10 min), detected only in low-temperature samples of *D. palmatum*, were characterized as *O*-acetyl-hexosides of luteolin and acacetin, respectively, according to UV and ESI-MS fragmentation patterns [54]. The presence of acylation in structures **20** and **25** was explained by their late elution compared with deacylated analogs, such as luteolin-7-*O*-glucoside (**13**; t_R_ 19.48 min) and acacetin-7-*O*-glucoside (**23**; t_R_ 36.72 min), respectively. The ESI-MS spectra in negative ion mode showed the peaks of the [M − H]^−^ ions and their deacylated fragments: 489→447 for **20** (Figure 1d) and 487→445 for **25**, which were useful for the characterization of the type of acyl group as an acetyl-group [53]. The presence of acetylated hexosides of luteolin and acacetin was not shown in *D. palmatum* previously, but has been described in *D. foetidum* [48] and *D. peregrinum* [49,50].

Five compounds were detected within the group of flavanone glycosides, and three of them were identified as eriodictyol-7-*O*-rutinoside (eriocitrin, **10**), eriodictyol-7-*O*-glucoside (**12**) and naringenin-7-*O*-glucoside (prunin, **16**) by comparison with standards. Eriodictyol-7-*O*-glucoside (**12**) and naringenin-7-*O*-glucoside (**16**) were found previously in *D. palmatum* [8] and *D. rupestre* [11]. Eriodictyol-7-*O*-rutinoside (**10**) was a new component for *D. palmatum* and in the *Dracocephalum* genus.

ESI-MS and UV spectra of components **6** and **9** were identical and lead readily to the determination of both compounds as flavanone-*O*-acyl-hexosides [54]. Based on *m*/*z* values, these flavonoids should be isomeric *O*-malonyl-hexosides of eriodictyol. UV and MS product spectra confirmed the identity of **6** and **9** as aglycone using an eriodictyol standard. The type of acyl group (malonyl) was confirmed by the presence of both acylated and deacylated fragments in ESI-MS spectra in negative ion mode (Figure 1c). Known literature regarding glycosides of eriodictyol showed no information about the *O*-malonyl-hexoside of eriodictyol, demonstrating that **6** and **9** are new isomeric natural compounds. Even though malonyl-glycosyl derivatives of flavonoids are rare components of the Lamiaceae family, they have already been isolated from *D. foetidum* [48], indicating the ability of the *Dracocephalum* plant to synthesize acylated flavonoid glycosides.

Nine flavonoid aglycones were detected in *D. palmatum* samples, including two flavanones (**21** and **26**) and seven flavones (**24**, and **27**–**32**). Eight of them were identified by comparing their UV and ESI-MS patterns with reference samples: eriodictyol (**21**), luteolin (**24**), naringenin (**26**), apigenin (**27**), chrysoeriol (**28**), acacetin (**29**), salvigenin (**31**) and genkwanin (**32**). Component **30** showed the [M − H]^−^ ion at *m*/*z* 329 and a specific UV spectrum with maxima at 302 and 330 nm, which are characteristic for isothymusin [55]. Four flavonoid aglycones (**21**, **24**, **26**, and **27**) were identified in wild samples of *D. palmatum* [8], while components **28**–**32** were discovered in this *Dracocephalum* species for the first time.

**Figure 2:**
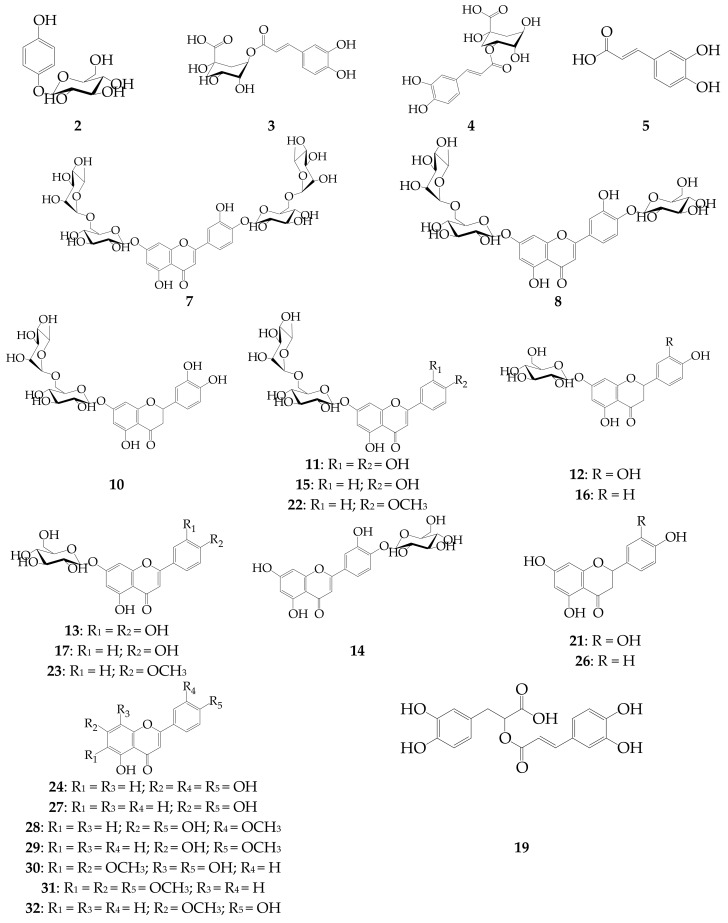
Structures of phenolic compounds **2**–**5**, **7**, **8**, **10**–**17**, **19, 21**–**24**, and **26**–**32** detected in *D. palmatum* herb.

These results suggest that the phenolic profile of *D. palmatum* may be successfully described using the online technique of RP-HPLC-DAD-ESI-MS, which allowed the characterization of 32 components (Figure 2). Although some of the phenolics (**4**, **5**, **7**, **8**, **11**–**17**, **19**, **21**, **24**, **26**, and **27**) have been previously described in *D. palmatum*, others (**1**–**3**, **10**, **18**, **20**, **22**, **23**, **25**, and **28**–**32**) have been discovered in this *Dracocephalum* species for the first time, including two new acylated flavanone glycosides (**6** and **9**).

Application of RP-HPLC-DAD for quantification of components **1**–**32** in *D. palmatum* showed that the composition of simple phenols, phenylpropanoids, flavone and flavanone aglycones and glycosides was significantly influenced by abiotic stress signals (e.g., cold-temperature conditions) (Table 5).

The amount of *O*-malonyl-arbutin (**1**) in a sample cultivated in NT conditions was 3.2 times lower than in cultivars grown in LT environments (1.24 mg/g). Arbutin content varied insignificantly in the both experimental groups. There were no qualitative differences in phenylpropanoid profiles of all experimental groups. The high amount of both caffeoylquinic acids (**3** and **4**) was noticed for the NT group. Their reduction was observed in LT conditions. However, the levels of caffeic (**5**) and rosmarinic acids (**19**) were the highest for LT samples and exceeded the amounts in samples from NT by 1.4 and 1.3 times, respectively. It should be noted that the high amount of **19** was noted earlier for *D. kotschyi* gathered from cold-temperature environments [56].

Significant changes in the flavonoid profiles of *D. palmatum* were observed. It has already been mentioned that flavones were represented by acacetin, apigenin and luteolin derivatives with the prevalence of the latter. Luteolin-7-*O*-glucoside (**13**) was the dominant flavonoid in NT group (2.56 mg/g) and LT group (29.56 mg/g). The highest amounts of other luteolin derivatives such as luteolin-7,4′-di-*O*-rutinoside (**7**), luteolin-7-*O*-rutinoside-4′-*O*-glucoside (**8**), luteolin-4′-*O*-glucoside (**14**) and luteolin-*O*-acetyl-hexoside (**20**) were noted for *D. palmatum* cultivated in LT environments. In addition, a significant increase in the apigenin derivatives apigenin-7-*O*-glucoside (**17**) and apigenin-*O*-hexoside (**18**) was observed under the same conditions. It is well known that flavonoid glycosides can be influenced by climate conditions such as temperature. Low temperature enhances total flavonoids, which act as shielding components and are responsible for protecting plants from damage as a result of enzymatic repair inhibition, combined with higher quantities of reactive oxygen species [57,58]. In the present experiment, induced biosynthesis of luteolin glycosides was assumed as a possible response. Furthermore, luteolin glycosides were shown to have higher antioxidant activity than their corresponding apigenin glycosides, underlining their importance in the cold-stress response [59].

The total amounts of minor components such as acacetin derivatives also increased to a maximum with decreasing temperature. It should be noted that acacetin-*O*-acetyl-hexoside (**25**) in NT conditions was not detected. The maximum amount of acacetin glycosides was observed in the LT group (1.41 mg/g) exceeded the amount of the same compounds in NT groups 2.5 times. Data in the literature indicate that the increased amount of methoxylated flavonoids can be paralleled with the increase in *O*-methyltransferase enzyme activity during cold acclimation [60].

The HPLC-detectable glycosides of flavanone types were represented by eriodictyol and naringenin derivatives. Eriodictyol-*O*-malonyl-hexosides (**6** and **9**) in NT conditions were not detected, but were revealed in cold-temperature environments. An increase in the amount of eriodictyol-7-*O*-glucoside (**12**) and naringenin-7-*O*-glucoside (**16**) in the LT group was found when compared to plants from NT.

Significant diversities in the flavone and flavanone aglycone profiles of *D. palmatum* grown in all temperature conditions were observed. Common aglycones such as apigenin (**27**) and luteolin (**24**) were detected in both experimental groups; however, the amount of luteolin in the LT plants exceeded the same amount in *D. palmatum* cultivated in NT conditions by more than 10 times.

In addition, the presence of rare methoxylated flavone aglycones such as acacetin (**29**), isothymusin (**30**), salvigenin (**31**) and genkwanin (**32**) was revealed only in plants cultivated in LT conditions. The sum of flavone aglycones in this group (14.6 mg/g) significantly exceeded the amount of aglycone compounds in NT environments (1.74 mg/g). Flavanone eriodictyol (**21**) was detected in the NT group in quantities less than 2.9 times compared to plants cultivated in LT conditions, while naringenin (**26**) was revealed only in the latter experimental group. Therefore, plants under LT are characterized by an increased concentration of free aglycones, including polymethoxylated flavonoids.

The high amount of lipophilic flavonoid aglycones can be explained by their ecophysiological role, which includes providing plants with protection from abiotic stress signals [61]. The lipophilic nature of flavonoid aglycones limited their distribution in plants, which is in contrast to water-soluble flavonoid glycosides. As they usually accumulate on the leaf surface and are extruded through the cuticle, they are known as surface or external flavonoids. Flavonoid aglycones, especially in the highly methylated form, accumulate in the Lamiaceae family [62]. The early note that species of the *Dracocephalum* genus are unable to accumulate external flavonoids as aglycons should be revised [63]. Low temperatures are known to positively affect the accumulation of the cuticular leaf wax. This mechanism protects plant tissues from cold stress [64]. Lipophilic flavonoid aglycons (polyhydroxylated and polymethoxylated) covalently bind to cutin or associate with waxes [65,66]. Thus, the presence of polymethoxylated flavonoid aglycones suggests its vital role as the first site of plant defense against abiotic stresses, such as LT. Thus, exposing *D. palmatum* plants to HT or LT resulted in significant changes in the phenolic composition of its metabolome.

### 2.5. Changes of Carbohydrates of D. palmatum during LT-Cultivation

**Figure 3:**
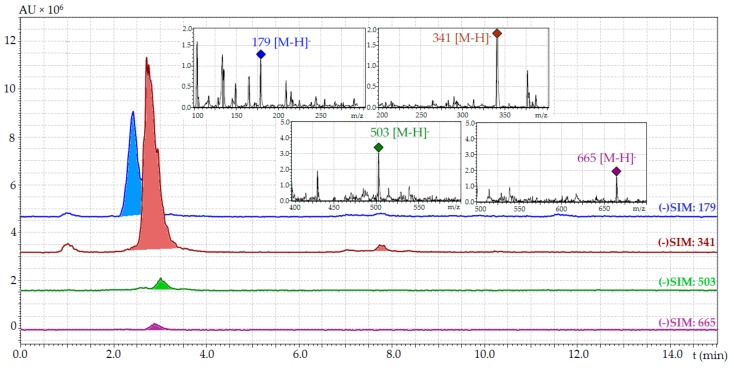
RP-HPLC-MS chromatograms in selected ion monitoring mode (SIM, negative ionization) of free sugars fraction of *D. palmatum* herb (low-temperature sample). SIM with following *m*/*z* value used: 179 for glucose, 341 for sucrose, 503 for raffinose, 665 for stachyose. On cuts, mass spectra of corresponding compounds; AU, absorbance units.

Carbohydrates of plants are key indicators of biochemical changes during temperature acclimation and the acquisition of cold tolerance [67]. Various forms of soluble sugars are involved in physiological reactions to temperature stress: simple sugars such as the mono- and oligosaccharides and polymeric components such as polysaccharides. Known data about soluble sugars of Lamiaceae display a wide distribution of glucose and sucrose as well as the raffinose family oligosaccharides (RFO) [68]. Application of RP-HPLC-MS in selected ion monitoring mode with negative ionization allowed detecting four carbohydrates in samples of *D. palmatum* (Figure 3).

Components were identified as glucose, sucrose (α-d-glucopyranosyl-(1→2)-β-d-fructofuranoside), raffinose (α-d-galactopyranosyl-(1→6)-α-d-glucopyranosyl-(1→2)-β-d-fructofuranoside) and stachyose (α-d-galactopyranosyl-(1→6)-α-d-galactopyranosyl-(1→6)-α-d-glucopyranosyl-(1→2)-β-d-fructofuranoside) using HPLC and ESI-MS-data compared to standards (Table 6). Sucrose accumulated to a much higher extent at all temperatures of *D. palmatum* cultivation (35.54–169.21 mg/g) (Table 6). The concentrations of raffinose (0.87–9.36 mg/g) and stachyose (1.72–38.95 mg/g) were lower than glucose (16.86–26.39 mg/g).

The levels of glucose and oligosaccharides in *D. palmatum* gradually increased during the low-temperature experiment. Total concentration of mono- and oligosaccharides in *D. palmatum* increased from 54.99 mg/g in group with NT cultivation to 243.91 mg/g in the group with a LT of cultivation. The differences between separate sugar amounts in the 20 °C and 1 °C groups were 1.6-, 4.8-, 10.8- and 22.6-fold for glucose, sucrose, raffinose and stachyose, respectively.

Previous studies have demonstrated that soluble low-molecular weight sugars play multiple roles in LT tolerance of plants. Concentrations of glucose and sucrose may increase several fold during exposure to LT [69]. The accumulation of sucrose in cane sugar supports their function as an osmoprotectant that stabilizes cellular membranes and maintains turgor [70]. The RFOs (raffinose, stachyose) are especially associated with cold hardiness, LT and dormancy [71]. Reaction of the simple sugar profile of *D. palmatum* LT cultivation was typical for cold-tolerant crops: increases in mono- and oligosaccharide levels were observed. No dramatic changes occurred in *D. palmatum* sugars after HT cultivation (35 °C).

Cold acclimation of plants also induced changes both in non-structural or reserve polysaccharides as starch or inulin and cell wall polysaccharides like pectin and pectin-associated polymers [72]. For characterization of polymeric sugars of the herb *D. palmatum*, the high-molecular polysaccharides were isolated by ethanol precipitation, followed by dialysis to yield 2.29% and 9.86% (referring to the dried herb of the 20 °C and 1 °C plant groups, respectively) raw water-soluble polysaccharides (RWSP) (Table 7).

The dialyzed RWSP contained a high amount of uronic acid, which increased from 43.57 in the 20 °C group to 46.16% in the 1 °C group and a low concentration of protein components (2.61–2.75%). All samples of RWSP gave a positive reaction with iodine and Yariv reagent, demonstrating the presence of starch and arabinogalactan-protein complexes, respectively. No reaction with either resorcinol and Fehling’s reagent indicated the absence of inulin and polymeric mannans. The polysaccharide fractions were composed of galacturonic acid, galactose, glucose and arabinose as main sugars with ratios of 4.1:2.6:1.4:1 and 4.4:2.7:1.0:1 for plants grown at NT and LT, respectively. Quantitatively minor components of RWSP were mannose, rhamnose, glucuronic acid and fucose and trace monomers such as xylose and ribose. Thus, the polysaccharide composition of *D. palmatum* seems to be composed of a mixture of starch, arabinogalactans and/or arabinogalactan–protein complexes and pectic components with high uronic amount.

Regarding monosaccharide composition of RWSP samples, two evident changes observed with the polysaccharide complex of *D. palmatum* growing at LT should be mentioned: (i) the amount of glucose in RWSP increased after cultivation temperature increased; and (ii) the amount of galacturonic acid, galactose and arabinose in RWSP increased after cultivation temperature decreased. The first event may be caused by the influence of the main plant glucose-containing polymer (starch), whose amount typically declines during exposure to LTs due to hydrolysis [73]. Cold-induced starch degradation was supported many times for various species as a process involved in the freezing tolerance enhancement of plants during an early phase of cold acclimation [74]. The cumulative effect of LT on galacturonic acid as a principal component of pectin and galactose and arabinose as monomers of satellite arabinogalactans was also previously discussed. Pectins appear to be a key element of the plant response to cold stress as shown by a number of studies on various species [75]. In *Pisum sativum* L., cold acclimation was accompanied by an increase in galacturonan and highly branched rhamnogalacturonan with branched and unbranched arabinans and galactans. The increased cold tolerance might be related to increased synthesis of arabinan and galactan side chains and galacturonan, which may act as a gelling component and a cold protectant [76].

### 2.6. Changes of Malondialdehyde, Antioxidant Enzymes and Antioxidant Potential of D. palmatum during LT-Cultivation

Low temperature not only caused various phenotypic changes but also initiates cellular damages reflected on increased electrolyte leakage and misbalance in reactive oxygen species (ROS) level and antioxidant content. Malondialdehyde (MDA) served as an indicator of cell membrane injury, elevated concentration of ROS and lipid peroxidative processes [77]. The level of MDA in NT plants was 92.74 nM/g fresh weight in opposite to LT plants with a value 197.02 nM/g fresh weight (Table 8). These results verified a significant raise in MDA content in leaf tissues after LT treatment, signifying that cold stress affected oxidative lipid injury. For scavenging of ROS, plants have a specific antioxidant mechanisms included enzymatic (superoxide dismutase, catalase and other) and non-enzymatic antioxidants [78]. Superoxide dismutase (SOD) plays a determinant role in protection against the toxic effects of oxidative stress by scavenging superoxide radicals and promoting their conversion into oxygen and hydrogen peroxide [79]. Catalase is an enzyme that functions in H_2_O_2_ degradation, which maintains hydrogen peroxide homeostasis in plants [80]. We measured the activities of two enzymatic antioxidants as superoxide dismutase and catalase in *D. palmatum* leaf tissue of NT an LT groups. The results indicated that the low temperature induced a significant increase of activity of both enzymes in 4.6 and 1.7 times comparing with NT plants (respectively, for SOD and catalase). The rising activity of antioxidant enzymes is a plant response to high level of the ROS, resulting in protection of cell membranes from increased MDA to accommodate LT stress [81].

The antioxidant properties of *D. palmatum* extracts were also analyzed by plenty methods both single-electron transfer (SET) and combination of SET with hydrogen-atom transfer techniques. To the latter methods total antioxidant capacity, DPPH^•^ (2,2-diphenyl-1-picrylhydrazyl) and ABTS^•+^ (2,2′-azino-bis(3-ethylbenzthiazoline-6-sulfonic acid) scavenging tests were related and successfully performed (Table 8). In turn among SET methods superoxide (O_2_^•−^) and bromine (Br^•^) radicals scavenging assays; nitric oxide (NO) and hydrogen peroxide (H_2_O_2_) inactivating tests and ferrous (II) ion (Fe^2+^) chelating assay were applied. In addition, all methods mentioned above were employed for evaluation of antioxidant potential of luteolin-7-*O*-glucoside, the dominant compound of *D. palmatum* herb with known property to prevent oxidation [82].

The total antioxidant capacity varied significantly in plant extracts and ranged from 280.98 mg/g (NT) to 682.26 mg/g (LT) (Table 8). In the DPPH^•^ and ABTS^•+^ assays, the LT plants demonstrated high efficiency in the scavenging of free radicals (IC_50_ 11.40 and 5.69 µg/mL, respectively).

The plants cultivated in NT conditions demonstrated less pronounced scavenging activity to inactivate DPPH^•^ and ABTS^•+^ free radicals. The scavenging value against superoxide radicals was highest in the group cultivated in LT environments (IC_50_ 9.21 µg/mL). The efficiency of luteolin-7-*O*-glucoside in this assay was lower (IC_50_ 14.92 µg/mL). The extract obtained from the herb grown in LT manifested significant scavenging activity of NO molecules (21.37 µg/mL). The herb extracts from plants cultivated in NT conditions demonstrated less pronounced scavenging activity (IC_50_ 37.92 µg/mL, respectively). In addition, the activity of the LT group in the H_2_O_2_ inactivating assay and Fe^2+^-chelating activity assay was characterized as very high in both methods, opposite of the low activity of luteolin-7-*O*-glucoside. It should be noted that herbal extract cultivated in the LT group was the most active antioxidant in all assays. Early information about the quantitative levels in the experimental groups allowed us to associate the significant antioxidant potential of this extract with the highest total phenolics levels.

Previously, the antioxidant properties of several *Dracocephalum* extractions were analysed. Thus, methanol extract from the aerial parts of *D. moldavica* revealed high efficiency in the scavenging of DPPH^•^, ABTS^•+^ and superoxide anion radicals (IC_50_ 23.10, 8.0 and 445.5 µg/mL, respectively). The total phenolic value was 289.55 mg/g of dry extract, and rosmarinic acid was the major polyphenol (107.11 mg/g of dry extract) [83]. Luteolin-7-*O*-glucoside isolated from whole plant *D. tanguticum* exhibited the highest antioxidant effect in DPPH^•^ (IC_50_ 3.94 µM), ABTS^•+^ (IC_50_ 56.46 µM) and ferrous ions radicals (IC_50_ 1.10 mM) [84]. The methanol extract of *D. heterophyllum* demonstrated high efficiency in the scavenging of DPPH^•^ radicals (IC_50_ 37.0 µg/mL) [85]. The total phenolic amount of the *D. kotschyi* methanol extract from leaves was 175.6 mg/g and DPPH^•^ scavenging capacity of the same extract was 88.99% [86]. The methanol extract from the aerial portion of *D. polychaetum* revealed effective scavenging of DPPH^•^ radicals (IC_50_ 5.6 mg/mL) [87].

Bioactivity of *D. palmatum* extracts was dependent from cultivation temperature level. In particular, the antiradical potential against various particles, including neutral 2,2-diphenyl-1-picrylhydrazyl radical and bromine radical, and charged 2,2′-azino-bis(3-ethylbenzthiazoline-6-sulfonic acid) cation-radical and superoxide anion-radical was the highest for the samples cultivated in cold-temperature condition. Inactivating power of the same plant extract in relation nitric (II) oxide and hydrogen peroxide molecules as well as Fe^2+^ chelating ability significantly exceeded the results obtained in other experimental groups and was similar to or exceeded the activity of the reference antioxidant, luteolin-7-*O*-glucoside.

## 3. Materials and Methods

### 3.1. Chemicals

The following chemicals were purchased from Biosupplies Australia Ply Ltd. (Victoria, Australia): Yariv reagent kit (Cat. No. 100-4); Extrasynthese (Lyon, France): acacetin-7-*O*-rutinoside (linarin; Cat. No. 1169, ≥98.5%); apigenin-7-*O*-rutinoside (isorhoifolin; Cat. No. 1121, ≥98.5%); 5-*O*-caffeoyl quinic acid (neochlorogenic acid; Cat. No. 4961, ≥99%); 3-*O*-caffeoylquinic acid (chlorogenic acid; Cat. No. 4991, ≥99%); caffeic acid (Cat. No. 6034, ≥99%); luteolin-7-*O*-glucoside (cynaroside; Cat. No. 1126, ≥98%); luteolin-4′-*O*-glucoside (Cat. No. 1083, ≥95%); rosmarinic acid (Cat. No. 4957, ≥99%); ChemFaces (Wuhan, China): acacetin-7-*O*-glucoside (tilianin; Cat. No. CFN92764, ≥95%); chrysoeriol (Cat. No. CFN98785, ≥95%); isothymusin (Cat. No. CFN97562, ≥95%); salvigenin (Cat. No. CFN99883, ≥95%); raffinose (Cat. No. CFN90425, ≥98%); stachyose (Cat. No. CFN90424, ≥98%); Santa Cruz Biotechnology, Inc. (Dallas, TX, USA): β-phellandrene (Cat. No. sc-477582, ≥98%); Sigma-Aldrich (St. Louis, MO, USA): acacetin (Cat. No. 00017, ≥97%); apigenin (Cat. No. 10798, ≥95%); apigenin-7-*O*-glucoside (cosmosiin, Cat. No. 44692, ≥97%); arabinose (Cat. No. A3256, ≥99%); arbutin (Cat. No. A4256, ≥98%); 2,2′-azino-bis(3-ethylbenzothiazoline-6-sulfonic acid) diammonium salt (Cat. No. A1888, ≥98%); bornyl acetate (Cat. No. 45855, ≥99%); camphene (Cat. No. 456055, ≥95%); β-caryophyllene (Cat. No. 75541, ≥98.5%); caryophyllene oxide (Cat. No. 91034, ≥99%); 1,8-cineol (Cat. No. 29210, ≥99%); *p*-cymene (Cat. No. 30039, ≥99.5%); *p*-cumenol (Cat. No. 175404, ≥98%); cuminaldehyde (Cat. No. 16679, ≥97%); 2,2-diphenyl-1-picrylhydrazyl (Cat. No. D9132); eriodictyol (Cat. No. 74565, ≥95%); eriodictyol-7-*O*-rutinoside (eriocitrin; Cat. No. 45714, ≥98%); eriodictyol-7-*O*-glucoside (Cat. No. 19474, ≥99%); Fehling’s reagent I (Cat. No. 36018); fucose (Cat. No. F8150, ≥98%); galactose (Cat. No. G0750, ≥99%); galacturonic acid monohydrate (Cat. No. 48280, ≥97%); genkwanin (Cat. No. SMB00422, ≥98%); glucose (Cat. No. G8270, ≥99.5%); glucuronic acid (Cat. No. G5269, ≥98%); isoamyl acetate (Cat. No. 79857, ≥99.7%); limonene (Cat. No. 62118, ≥99%); linalool (Cat. No. 51782, ≥99%); lithium perchlorate (Cat. No. 431567, ≥99.99%); luteolin (Cat. No. L9283, ≥98%); luteolin-7-*O*-rutinoside (scolymoside; Cat. No. SMB00200, ≥95%); mannose (Cat. No. M8574, ≥99%); β-myrcene (Cat. No. W276200, ≥95%); myrtenyl acetate (Cat. No. 80699, ≥95%); naringenin (Cat. No. N58ra93, ≥95%); naringenin-7-*O*-glucoside (prunin; Cat. No. SMB00076, ≥95%); α-pinene (Cat. No. 80605, ≥98.5%); β-pinene (Cat. No. 80609, ≥98.5%); trans-pinocarveol (Cat. No. 80613, ≥96%); perchloric acid (Cat. No. 311421, ≥70%, 99.999% trace metals basis); resorcinol (Cat. No. 398047, ≥99%); ribose (Cat. No. R7500, ≥99%); rhamnose (Cat. No. W373011, ≥99%); sabinene (Cat. No. 275166, ≥99%); sucrose (Cat. No. S9378, ≥99.5%); γ-terpinene (Cat. No. 86476, ≥98.5%); terpinene-4-ol (Cat. No. 49598, ≥95%); and xylose (Cat. No. X1500, ≥99%). Luteolin-7,4′-di-*O*-rutinoside (dracopalmaside) and luteolin-7-*O*-rutinoside-4′-*O*-glucoside (cynarotriside) were isolated previously from *D. palmatum* [9].

### 3.2. Plant Material, Growth Conditions, and Stress Treatments

*D. palmatum* were grown from authenticated seeds obtained from Tsitsin’s Main Botanical Garden of the Russian Academy of Science (Moscow, Russia). Seeds were collected from the Republic Yakutia, Kobyaiskii region, village Kitchan (year of collection 1991). Seeds were sterilized by incubation for 1 min in 75% ethanol and then washed thoroughly with sterile water. The seeds were germinated in soil in peat pots at 20 °C (16 h light at photon flux 135 µmol m^−2^ s^−1^/8 h dark at 16 °C). Seedlings at the age of two-month old were subjected to environmental stress. Plants were divided into two groups, one group continued to grow in normal temperature conditions (20 °C). For low temperature treatments, seedlings were transferred to a temperature of 1 °C in an artificial climate box for 20 days. The following light and photoperiodic conditions have been used for seedlings growing: 16 h light at 20 °C (or 1 °C) and photon flux 490 µmol m^−2^ s^−1^/8 h dark at 20 °C (or 1 °C). The herbs were collected after 20 days of the environmental stress treatment. The experimental samples were immediately frozen in liquid nitrogen and stored at −80 °C until use for analysis of photosynthetic pigments, malondialdehyde and enzyme activity. A part of fresh material was shade dried at room temperature before analytical assays (phenolic compounds, free sugars), isolation procedures (fatty acids, essential oil, polysaccharides) and bioactivity determination (dry extracts).

### 3.3. Electrolyte Leakage

Electrolyte leakage was measured following method of Campos et al. [88]. Twenty freshly cut leaf discs (0.5 cm^2^ each) were rinsed 3 times (2–3 min) with demineralized water and subsequently floated at 4 °C on 10 mL of demineralized water in the dark in the growth chamber. The conductivity of the suspending solution was measured after 24 h using an electrical conductivity analyzer (Expert-002-2-6n; Econics-Expert Co., Ltd., Moscow, Russia) before and after autoclaving at 120 °C for 30 min to release the total electrolytes. Electrolyte leakage was calculated as a percentage of total electrolytes. The results were presented as the mean values ± SD (standard deviations) of three replicates.

### 3.4. Photosynthetic Pigments Analysis

Photosynthetic pigments (chlorophylls, carotenes, pheophytins) were determined using multi-wavelength spectrophotometric method. Frozen weighed plant leaves (0.5 g) were homogenized under liquid nitrogen and an aliquot of 2 g was weighed into a 50-mL centrifuge tube with 10 mL of acetone/water mixture (4:1). After vortexing (1 min), mixing, and shaking for 30 min, samples were centrifuged for 15 min at 3000× *g* at room temperature. The procedure of acetone/water extraction was repeated twice and organic fractions were combined in a volumetric flask (50 mL). The final volume of organic extract was reached to 50 mL by acetone/water mixture (4:1). The absorbance of the organic extract was measured using SF-2000 UV-Vis-spectrophotometer (OKB Specter; St. Petersburg, Russia) in a 1 cm quartz cuvette at 470/646.8/663.2 nm (chlorophylls, carotenes) and 536/666 nm (pheophytins) blanked with acetone/water mixture (4:1). Pigment concentrations were calculated using corresponding absorption coefficients [89,90]. The results were expressed in fresh weight basis and were presented as the mean values ± SD (standard deviations) of three replicates.

### 3.5. Chlorophyll Fluorescence and Carbon Assimilation Rate Measurement

Chlorophyll fluorescence was measured on the upper leaf surfaces by PAM fluorometer Junior PAM (Heinz Walz GmbH, Effeltrich, Germany). The following fluorescence parameters were measured: minimal fluorescence in the dark-adapted state (F_0_) and the maximal fluorescence in the dark adapted state (F_m_). Maximal quantum yield of PSII photochemistry (F_v_/F_m_) was calculated according to equation: F_v_/F_m_ = (F_m_ − F_0_)/F_m_. The parameter of the carbon assimilation rate (as µM CO_2_/m^2^·s) was analyzed using infrared gas analyzer LCpro+ Portable Gas Exchange System (ADC BioScientific Ltd., Hertfordshire, UK) coupled with Small Leaf Chamber. The results were presented as the mean values ± SD (standard deviations) of ten replicates.

### 3.6. Fatty Acids Analysis

Fatty acids were extracted using a modified Folch method [91] following by the esterification into fatty acids methyl esters (FAMEs) [92]. The pulverized dried herb (0.1 kg) was extracted at 70 °C for 10 h by Soxhlet extractor with 500 mL of chloroform-methanol (2:1) mixture. The solvent was evaporated to dryness at 35 °C and to yield the fatty acid fraction (FAF). Samples of FAF (0.1 g) were dissolved in 4 mL of 0.5 N sodium hydroxide solution in methanol and boiled under reflux for 60 min. After saponification, 3 mL of boron trifluoride methanolic solution (14%, *v*/*v*) was added and the solution boiled for 15 min following by hexane (6 mL) addition and heating under reflux (10 min). Then saturated sodium chloride (1 mL) was added, vortexed (10 s) and centrifuged at 6000 rpm (15 min). The upper hexane layer was removed, concentrated (N_2_), and the resultant residue was dissolved in hexane (200 µL) before gas chromatography-mass spectrometry (GC-MS) analysis.

The GC-MS system was consisted of an Agilent 6890 N gas chromatograph and an Agilent Technologies 5973 N mass selective/quadrupole detector (Agilent Technologies Inc., Santa Clara, CA, USA). A capillary column HP-INNOWax with a length of 30 m, inner diameter of 0.25 mm, and film thickness of 0.5 µm was used for separation (Agilent Technologies Inc., Santa Clara, CA, USA) with helium at a 25 mL/min as a carrier gas. The temperature was 40 °C after injection then programmed at 2 °C/min to 300 °C, and maintained at that temperature for 45 min. A solvent delay of 9 min was set before MS acquisition began. The transfer line from GC column to MS was set to 180 °C, the source 230 °C, and the quadrupole 150 °C. MS detector was done by electron ionization at 70 eV, with a scan range of 30 a.m.u. to 700 a.m.u. (1 scan/s). Compounds were identified by using online NIST 05 and Wiley-7th library spectra, published MS data and analytical standards parameters of available FAMEs. Stock standard solutions of available FAMEs were prepares by dissolving FAME mixture (Supelco-37 mixture C_4_–C_24_, Sigma-Aldrich, St. Louis, MO, USA) in hexane at a concentration 0.2 mg/mL. Tridecanoic acid was used as the internal standard and was dissolved in hexane at concentration 0.5 mg/mL. The relative content of each FAME was calculated by normalization of the obtained total ion current peak areas as the percentages of total fatty acids.

### 3.7. Essential Oil Analysis

The pulverized dried herb (300 g) was placed in a 2000 mL round-bottom flask along with 600 mL distilled water and subjected to Clevenger hydrodistillation for 2.5 h. The oil was extracted from the distillate with hexane and then dried over anhydrous sodium sulfate. The solvent was evaporated using rotary evaporator gave the essential oil. The aliquot of essential oil (10 µL) was dissolved in hexane (500 µL) before gas chromatography-mass spectrometry (GC-MS) analysis.

The GC-MS system was consisted of an Agilent 6890 N gas chromatograph and an Agilent Technologies 5973 N mass selective/quadrupole detector (Agilent Technologies Inc., Santa Clara, CA, USA). A capillary column HP-5MS with a length of 30 m, inner diameter of 0.25 mm, film thickness of 0.5 µm and 5% diphenyl- and 95% dimethylpolysiloxane as stationary phase was used for separation (Agilent Technologies Inc., Santa Clara, CA, USA) with helium at a 1 mL/min as a carrier gas. The temperature was 150 °C after injection then programmed at 2 °C/min to 250 °C, and maintained at that temperature for 45 min. A solvent delay of 9 min was set before MS acquisition began. The transfer line from GC column to MS was set to 250 °C, the source 230 °C, and the quadrupole 150 °C. MS detector was done by electron ionization at 70 eV, with a scan range of 41 a.m.u. to 450 a.m.u. (1 scan/s). Compounds were identified by using online NIST 05 and Wiley-7th library spectra, published MS data and analytical standards parameters of available compounds. Stock standard solutions of available aliphatic compounds, phenolic and tepenes were prepared by dissolving each compound in hexane separately at a concentration 0.5 mg/mL. Decane was used as the internal standard and was dissolved in hexane at concentration 1.0 mg/mL. Component relative percentages were calculated based on normalization method without using correction factors.

### 3.8. Phenolic Compounds Analysis

Reversed-phase high-performance liquid chromatography with diode array detection and electrospray ionization mass spectrometry (RP-HPLC-DAD-ESI-MS) procedure was used for phenolic compounds qualitative and quantitative analysis. Sample preparation for RP-HPLC-DAD-ESI-MS analysis: an accurately weighted, dried, and powdered *D. palmatum* herb samples (200 mg) were placed in a conical flasks. Then 5 mL of 60% methanol were added and the mixtures were weighted. The samples were then extracted in an ultrasonic bath for 60 min at 45 °C with an ultrasound power of 100 W and frequency of 35 kHz. After cooling, the flasks weights were reduced to initial sign, and the resultant extracts were filtered through a 0.22-µm polytetrafluoroethylene (PTFE) syringe filter before injection into the HPLC system for analysis.

Experiments were performed on an LCMS 8050 liquid chromatograph coupled with diode-array-detector and triple-quadrupole electrospray ionization detector (Shimadzu, Columbia, MD, USA), using a GLC Mastro C18 column (150 × 2.1 mm, Ø 3 µm; Shimadzu, Kyoto, Japan); the column temperature was 30 °C. Eluent A was water and eluent B was acetonitrile. The injection volume was 1 µL, and elution flow was 200 µL/min. Gradient program: 0–5 min, 5–18% B; 5–12 min, 18–20% B; 12–25 min, 20% B; 25–37 min, 20–45% B; 37–43 min, 45–70% B; 43–58 min, 70–100% B; and 58–60 min, 100–5% B. The DAD acquisitions were performed in the range of 200–600 nm and chromatograms were integrated at 280 nm. For ESI-MS, the parameters were set as follows: temperature levels of ESI interface, desolvation line and heat block were 300 °C, 250 °C and 400 °C, respectively; the flow levels of nebulizing gas (N_2_), heating gas (air) and collision-induced dissociation gas (Ar) were 3 L/min, 10 L/min and 0.3 mL/min, respectively. The capillary voltage was kept at +4 kV (simple phenols) in positive mode and at −4.5 kV (phenylpropanoids and flavonoids) in negative mode. ESI-MS spectra were recorded by scanning in the range of *m*/*z* 100–1900.

Quantification of phenolic compounds was realized in RP-HPLC-DAD experiments using chromatographic conditions mentioned above. To prepare the stock solutions of reference compounds, 1 mg each of arbutin, 5-*O*-caffeoylquinic acid, 3-*O*-caffeoylquinic acid, caffeic acid, luteolin-7,4′-di-*O*-rutinoside (dracopalmaside), luteolin-7-*O*-rutinoside-4′-*O*-glucoside (cynarotriside), eriodictyol-7-*O*-rutinoside (eriocitrin), luteolin-7-*O*-rutinoside (scolymoside), eriodictyol-7-*O*-glucoside, luteolin-7-*O*-glucoside (cynaroside), luteolin-4′-*O*-glucoside, apigenin-7-*O*-rutinoside (isorhoifolin), naringenin-7-*O*-glucoside (prunin), apigenin-7-*O*-glucoside (cosmosiin), rosmarinic acid, eriodictyol, acacetin-7-*O*-rutinoside (linarin), acacetin-7-*O*-glucoside (tilianin), luteolin, naringenin, apigenin, chrysoeriol, acacetin, isothymusin, salvigenin, and genkwanin were accurately weighed and individually dissolved in DMSO/methanol mixture (1:4) in volumetric flasks (1 mL). The external standard calibration curve was generated using five data points, covering the concentration ranges 0.25–1.00 mg/mL. The calibration curves were created by plotting the peak area vs. the concentration levels. Taxifolin (t_R_ 32.54 min) and gardenin B (t_R_ 48.63 min) were used as the internal standards and was dissolved separately in DMSO/methanol mixture (1:4) at concentration 1 mg/mL. The concentrations of *O*-malonyl-arbutin, eriodictyol-*O*-malonyl-hexosides, apigenin-7-*O*-hexoside, luteolin-7-*O*-acetyl-hexoside and acacetin-7-*O*-acetyl-hexoside were calculated using arbutin, eriodictyol-7-*O*-glucoside, apigenin-7-*O*-glucoside, luteolin-7-*O*-glucoside and acacetin-7-*O*-glucoside, respectively, as reference compounds. The following correction coefficients were used to calculate contents of *O*-malonyl-arbutin, eriodictyol-*O*-malonyl-hexosides, luteolin-7-*O*-acetyl-hexoside and acacetin-7-*O*-acetyl-hexoside: 1.316, 1.191, 1.094 and 1.094, respectively. All analyses were carried out in triplicate and the data were expressed as mean value ± standard deviation (SD).

### 3.9. Carbohydrates Analysis

#### 3.9.1. RP-HPLC-MS Analysis of Free Sugars

RP-HPLC-DAD-ESI-MS procedure was used for analysis of free sugars. Sample preparation for free sugar analysis: an accurately weighted, dried, and powdered *D. palmatum* herb samples (100 mg) were placed in a conical flasks. Then, 10 mL of 40% methanol were added and the mixtures were weighted. The samples were then extracted in an ultrasonic bath for 30 min at 50 °C with an ultrasound power of 100 W and frequency of 35 kHz. After cooling, the flasks weights were reduced to initial sign, and the resultant extracts were filtered through a 0.22-µm polytetrafluoroethylene (PTFE) syringe filter before injection into the HPLC system for analysis.

Experiments were performed on an LCMS 8050 liquid chromatograph (Shimadzu, Columbia, MD, USA) coupled with diode-array-detector (DAD) and triple-quadrupole electrospray ionization detector (ESI), using a GLC Mastro C18 column (150 × 2.1 mm, Ø 3 µm; Shimadzu, Kyoto, Japan); the column temperature was 25 °C. Eluent A was water and eluent B was acetonitrile–water mixture (20:80). The injection volume was 1 µL, and elution flow was 150 µL/min. Gradient program: 0–10 min, 2–8% B; and 10–16 min, 8–12% B. For selected ion monitoring mode (SIM) with negative ionization mass-spectrometry, the parameters were set as follows: SIM *m*/*z* values—179 a.m.u. for glucose, 341 a.m.u. for sucrose, 503 a.m.u. for raffinose, 665 a.m.u. for stachyose; temperature levels of ESI interface, desolvation line and heat block were 300 °C, 250 °C and 400 °C, respectively; the flow levels of nebulizing gas (N_2_), heating gas (air) and collision-induced dissociation gas (Ar) were 3 L/min, 10 L/min and 0.3 mL/min, respectively. The capillary voltage was kept at −4 kV in negative mode. RP-HPLC-MS quantification experiments were carried out at the same chromatographic conditions in SIM mode.

To prepare the stock solutions of reference sugars, 1 mg of glucose, sucrose, raffinose and stachyose were accurately weighed and individually dissolved in water in volumetric flasks (2 mL). The external standard calibration curve was generated using five data points, covering the concentration range 0.1–0.5 mg/mL. The calibration curves were created by plotting the peak area vs. the concentration levels. Quinic acid (t_R_ 9.26 min) was used as the internal standard and was dissolved in water at concentration 0.4 mg/mL. All the analyses were carried out in triplicate and the data were expressed as mean value ± standard deviation (SD).

#### 3.9.2. Raw Water Soluble Polysaccharide (RWSP) Analysis

Powdered dried *D. palmatum* herb (100 g) was extracted with 10 L of distilled water at 90 °C for 60 min. The water extract was filtered to remove plant material, concentrated in vacuo to 200 mL and finally centrifuged at 3000 rpm (20 min). The resultant supernatant was precipitated with 95% ethanol (1:5) and centrifuged at 3000 rpm (20 min). The crude polysaccharide pellets were redissolved in water (200 mL) and treated by Sevag method three times to remove protein contaminants [93]. Deproteinised polysaccharide solution was intensively dialyzed against distilled water in dialysis tubes (MW-cut off 2 kDa; Sigma-Aldrich, St. Louis, MO, USA). Finally non-dialysed solution was passed though cation-exchange resin KU-2-8 (H^+^-form, 200 g; Closed Joint-Stock Company Tokem, Kemerovo, Russia) to remove mineral contaminants. The volume of the polysaccharide solution was reduced in vacuo (200 mL) and liophylized using freeze-dry apparatus Scientz Ordinary 10N (Ningbo Scientz Biotechnology Co., Ltd., Ningbo, China) to obtain off-white powder of RWSP.

Total carbohydrate content in RWSP was determined by a modified anthrone-H_2_SO_4_ spectrophotometric assay with glucose as a standard [94]. Estimation of uronic acid content was realized using 3,5-dimethylphenol-H_2_SO_4_ spectrophotometric assay with galacturonic acid as a standard [95]. To determine the protein concentration in RWSP the Bradford colorimetric method was used with BSA as a standard [96]. The tests of RWSP with iodine, resorcinol-HCl reagent, Yariv reagent and Fehling’s reagent to qualify the presence of starch, inulin, arabinogalactan-protein complexes and mannans were performed as described previously [97,98,99].

The monosaccharide composition of RWSP was determined after acidic hydrolysis with trifluoroacetic acid (TFA) following by 1-phenyl-3-methyl-5-pyrazolone (PMP) labeling and microcolumn HPLC with ultraviolet detection separation (HPLC-UV) of PMP-labeled hydrolyzates. To hydrolyze RWSP samples 10 mg was dissolved in 1 mL of 2 M TFA in a 5 mL ampoule, incubated at 120 °C for 2 h, the cooled reaction mixture was centrifuged at 2000 rpm for 5 min and evaporated to dryness under reduced pressure to remove TFA, and the hydrolyzed and dried samples were redissolved in 1 mL of distilled water for the following experiments. PMP-labeling was performed using Sun et al. protocol [100] with slight modification. The hydrolyzed RWSP samples (50 µL) were labeled by adding 15 µL of NaOH (1.5 M) and 75 µL of PMP solution (0.5 M in methanol). The mixtures were incubated at 70 °C for 2 h, cooled to room temperature, and neutralized with 60 µL of HCl (0.5 M), 1 mL of chloroform was added, and, after vigorous shaking (20 s) and centrifuging at 3000 rpm for 10 min, the organic phase was carefully removed and discarded. The aqueous layer was passed through a 0.45 µm syringe filter before HPLC analysis.

Microcolumn HPLC-UV chromatographic separation of PMP-labeled sugars were achieved on an MiLiChrom A-02 microcolumn chromatograph (Econova; Novosibirsk, Russia) equipped with UV-detector. A microcolumn ProntoSIL-120-5-C18 AQ with a length of 75 mm, inner diameter of 1 mm, and particles diameter of 1 µm was used for separation (Metrohm AG, Herisau, Switzerland). Separation was performed in a gradient mode with 100 mM CH_3_COONH_4_ solution (pH 6.9) as eluent A, and acetonitrile as eluent B using the following gradient program: 0–20 min, 20–26% B. The column temperature was 35 °C, injection volume was 1 µL, and elution was at 150 µL/min. Chromatograms were recorded at 250 nm. The retention times of PMP-labeled monosaccharides were followed: 6.94 min for mannose, 8.94 min for ribose, 9.15 min for rhamnose, 10.91 min for glucuronic acid, 11.38 min for galacturonic acid, 13.11 min for glucose, 13.73 min for galactose, 14.93 min for xylose, 15.11 min for arabinose, and 16.90 min for fucose.

To prepare the stock solutions of reference sugars, 1 mg of mannose, ribose, rhamnose, glucose, galactose, xylose, arabinose, fucose, galacturonic acid and glucuronic acid were accurately weighed and individually dissolved in water in volumetric flasks (2 mL). All sugar reference samples were PMP-labeled before analysis. The external standard calibration curve was generated using five data points, covering the concentration range 0.01–0.05 mg/mL. The calibration curves were created by plotting the peak area vs. the concentration levels. All the analyses were carried out in triplicate and the data were expressed as mean value ± standard deviation (SD).

### 3.10. Antioxidant Activity Assays

Frozen leaves (2 g) were homogenized with 10 mL of 0.05 M sodium phosphate buffer solution (pH 7.8) with 1% polyvivylpyrrolidone and was centrifuged at 12,000× *g* at 4 °C for 15 min. The supernatant was collected for malondialdehyde (MDA) and enzyme analysis. MDA content was measured using spectrophotometric assay based on the thiobarbituric acid reaction [101]. Superoxide dismutase activity was analyzed using spectrophotometric method of Giannopolitis-Ries with measurements of the inhibition levels of nitroblue tetrazolium reduction at 560 nm [102]. Catalase activity was measured using spectrophotometric method of Abassi et al. which include the measuring of H_2_O_2_ extinction decline at 240 nm [103].

The total antioxidant capacity of *D. palmatum* extracts was determined using the phosphomolybdic acid method [104]; the DPPH^•^ radical scavenging activity and ABTS^•+^ radical scavenging activity was assessed as described early [105]; the determination of superoxide anion scavenging activity (O_2_^•−^-SA) was measured in phenazine methosulfate-nicotinamide adenine dinucleotide-nitroblue tetrazolium systems using the method of Ozen et al. [106]; the Br^•^ radical scavenging activity (Br^•^-SA) was determined using culometric method [105] with electrogenerated bromine radicals; the potential to inactivate NO was assessed by the sodium nitropusside technique [107]; the ability to inactivate H_2_O_2_ was determined using the technique developed by Badami and Channabasavaraj [108]; to evaluate the chelating activity for Fe^2+^ ions the *o*-phenanthroline technique was applied [59].

For preparation of the extract, accurately-weighed dried sample of *D. palmatum* herb (10 g) was transferred in a conical flask for preparation of the extracts. After that, 150 mL of solvent (60% methanol solutions) was added with stirring and put in an ultrasonic bath. The extraction conditions were 60 min at 45 °C, ultrasound power of 100 W, the frequency 35 kHz. The extraction was repeated twice. The obtained extract were filtered through a cellulose filter and combined. The filtrates were evaporated in vacuo until dryness with the use of a rotary evaporator.

### 3.11. Statistical Analysis

The results were given as the mean ± standard deviation (SD). One-way analysis of variance (ANOVA) with a post hoc least significant difference test was used to determine significance (*p* ≤ 0.05) with Statistica 5.5 software (Dell Technologies Inc., Round Rock, TX, USA) together with the correlation matrix with the use of elementary statistics.

## 4. Conclusions

Since *D. palmatum* is a typical plant of arctic territory of Yakutia, it is a suitable model plant to qualify and quantify the plant physiological and biochemical responses to temperature change by lipophilic and hydrophilic compound formation and bioactivity variation. In the past, such studies have not been carried out using Yakutian plant as an experimental object. The present study demonstrated that long-term cultivation (20 days) of *D. palmatum* seedlings at low temperature (1 °C) damaged plant organ integrity that affected on the rising level of electrolyte leakage and reactive oxygen species misbalance resulting in increasing concentration of malondialdehyde and antioxidant enzyme (superoxide dismutase and catalase). However, at the same time, accumulation of photosynthetic pigments, unsaturated fatty acids, EO and volatile monoterpenes; all groups of phenolic compounds including simple phenol derivatives, phenylpropanoids, flavone and flavanone derivatives; and soluble mono- and oligosaccharides and polysaccharides was observed in *D. palmatum.* All investigated antioxidant assays showed increased levels of bioactivity in the plant material collected at low temperature. For the first time, such multi-factor approach was used to research of *D. palmatum* and arctic plants as whole. While the relationship between temperature of plants cultivation and their chemical composition is complex, in the present study it was shown that the low temperature may cause specific chemical shifts achieving sustainable growth of the arctic plant. The scope and diverse of the information received allow to expand our knowledge about adaptive potential of arctic plants and their cold acclimation mechanisms. As far as the practical side is concerned, it is worth mentioning that the cold-induced accumulation of the various groups of compounds in *D. palmatum* confirmed the potential of low temperature cultivation for enrichment of arctic plants used in food and medicinal industries by bioactive components.

## Figures and Tables

**Table 1:** Electrolyte leakage (percent of total electrolytes ± SD), photosynthetic pigments content (µg/g of fresh leaf weight (FW) ± standard deviation (SD)), carbon assimilation rate (µM CO_2_/m^2^·s ± SD) and effective quantum yield of PSII (F_v_/F_m_; ±SD) in *D. palmatum* leaves cultivated under normal (20 °C) and low (1 °C) temperatures. ^1^

Parameter	Temperature (°C)	
	20	1
Electrolyte leakage, % of total electrolytes	18.2 ± 0.9 ^a^	26.7 ± 1.4 ^a^
Chlorophyll a content (Chl_a_), µg/g FW	273.45 ± 9.29 ^a^	406.19 ± 13.40 ^a^
Chlorophyll b content (Chl_b_), µg/g FW	75.85 ± 2.50 ^a^	141.21 ± 4.79 ^a^
Total chlorophylls content (ΣChl), µg/g FW	349.30	547.40
Chl_a_/Chl_b_	3.61	2.88
Pheophytin a content, µg/g FW	6.01 ± 0.16 ^a^	7.06 ± 0.14 ^a^
Pheophytin b content, µg/g FW	4.57 ± 0.10 ^a^	5.64 ± 0.11 ^a^
Total pheophytins content, µg/g FW	10.58	12.70
Carotenoids content (Car), µg/g DW	36.86 ± 1.07 ^a^	53.31 ± 1.55 ^a^
ΣChl/Car	9.48	10.27
Carbon assimilation rate, µM CO_2_/m^2^·s	8.3 ± 0.8 ^b^	5.8 ± 0.4 ^b^
F_v_/F_m_	0.62 ± 0.04 ^b^	0.54 ± 0.03 ^b^

^1^ Averages ± standard deviations were obtained from three (^a^) or ten (^b^) different experiments.

**Table 2:** Fatty acids (FA) composition of *D. palmatum* herb under different temperatures of cultivation (percentage of total FA content).

Compound	Temperature (°C)	
	20	1
Pelargonic acid (9:0)	0.1	Tr.
Capric acid (10:0)	0.2	Tr.
Lauric acid (12:0)	5.7	3.2
Tridecylic acid (13:0)	1.4	0.5
Myristic acid (14:0)	2.7	1.7
Pentadecylic acid (15:0)	0.4	0.3
Palmitic acid (16:0)	27.9	11.2
Palmitoleic acid (16:1)	3.8	6.9
Margaric acid (17:0)	1.8	1.4
Stearic acid (18:0)	8.0	5.2
Oleic acid (18:1 _ω-9_)	11.3	18.3
Linoleic acid (18:2 _ω-6_)	14.1	19.3
α-Linolenic acid (18:3 _ω-3_)	12.5	18.2
γ-Linolenic acid (18:3 _ω-6_)	2.1	7.4
Arachidic acid (20:0)	4.5	0.5
Gondoic acid (20:1 _ω-9_)	2.0	3.7
Behenic acid (22:0)	0.5	Tr.
Erucic acid (22:1 _ω-9_)	0.7	1.2
Lignoceric acid (24:0)	0.1	Tr.
Total	99.8	99.0
Saturated FA	53.3	24.0
Unsaturated FA	46.5	75.0
Monounsaturated FA	17.8	30.1
Polyunsaturated FA	28.7	44.9

Tr., traces (<0.1%).

**Table 3:** Essential oil (EO) composition (percentage of total component content) of *D. palmatum* herb under different temperatures of cultivation.

Compound	RI	MI ^a^	Temperature (°C)	
			20	1
Aliphatic compounds				
Isoamyl acetate	875	i, ii, iii	0.6	0.5
Subtotal			0.6	0.5
Simple phenols				
*p*-Cymene	1024	i, ii, iii	1.7	1.8
*p*-Cymene-8-ol	1186	i, ii	1.9	2.0
*p*-Cumenol	1222	i, ii, iii	0.4	0.4
*m*-Cumenol	1225	i, ii	0.1	0.1
Cuminaldehyde	1241	i, ii, iii	1.2	1.2
Subtotal			5.3	5.5
Monoterpenes				
α-Thujene	926	i, ii	0.6	0.4
α-Pinene	932	i, ii, iii	1.1	1.2
Camphene	947	i, ii, iii	0.1	0.2
Sabinene	973	i, ii, iii	2.5	2.0
β-Pinene	975	i, ii, iii	8.6	9.0
β-Myrcene	991	i, ii, iii	0.6	0.2
Pseudolimonene	1003	i, ii	0.3	0.2
β-Phellandrene	1027	i, ii, iii	4.8	5.2
Limonene	1029	i, ii, iii	1.8	1.8
1,8-Cineol	1031	i, ii, iii	5.5	5.8
γ-Terpinene	1058	i, ii, iii	0.2	0.3
Linalool	1100	i, ii, iii	1.2	1.4
β-Pinone	1105	i, ii	0.8	1.0
trans-Pinocarveol	1138	i, ii, iii	1.0	1.4
trans-Pinocamphone	1161	i, ii	37.9	40.7
*cis*-Pinocamphone	1175	i, ii	8.0	8.7
*cis*-Pinocarveol	1186	i, ii	0.2	0.5
Terpinene-4-ol	1177	i, ii, iii	0.5	0.4
Myrtenol	1197	i, ii	2.7	3.3
Phellandral	1276	i, ii	0.2	0.1
Bornyl acetate	1287	i, ii, iii	0.3	0.2
trans-Pinocarvyl acetate	1301	i, ii	1.8	2.0
*cis*-Pinocarvyl acetate	1315	i, ii	0.4	0.7
Myrtenyl acetate	1327	i, ii, iii	3.5	3.7
*p*-Mentha-1,4-dien-7-ol	1329	i, ii	0.4	0.1
Subtotal			85.0	90.8
Sesquiterpenes				
β-Caryophyllene	1420	i, ii, iii	1.0	0.4
γ-Cadinene	1518	i, ii	0.5	Tr.
Germacrene B	1560	i, ii	0.7	0.1
Caryophyllene oxide	1587	i, ii, iii	1.8	0.5
Viridiflorol	1594	i, ii	3.2	1.6
α-Cadinol	1659	i, ii	1.2	0.3
Germacrone	1696	i, ii	0.6	0.2
Subtotal			9.0	3.1
Total			99.9	99.9

^a^ Methods of identification: i, retention index; ii, mass spectrum; iii, co-injection with authentic sample. Tr., traces (<0.1%).

**Table 4:** HPLC parameters, ultraviolet spectra data (UV) and electrospray ionization mass spectrometry (ESI-MS) data of components **1**–**32** from *D. palmatum* herb.

No.	Compound	t_R_ (min)	UV, λ_max_ (nm)	ESI-MS (*m*/*z)*	Refs. Comp.^a^
**1**	*O*-Malonyl-arbutin	6.79	280	381 [M + Na]^+^, 352 [M + H]^+^, 295 [M + Na]^+^	iii [45]
**2**	Arbutin	7.89	280	295 [M + Na]^+^, 273 [M + H]^+^	i
**3**	5-*O*-Caffeoylquinic acid	10.43	331	353 [M − H]^−^, 183	i
**4**	3-*O*-Caffeoylquinic acid	12.03	331	353 [M − H]^−^, 183	i
**5**	Caffeic acid	13.15	323	179 [M − H]^−^	i
**6**	Eriodictyol-*O*-malonyl-hexoside (isomer)	14.09	284	535 [M − H]^−^, 449 [(M − malonyl) − H]^−^, 287 [(M − malonyl − Hex) − H]^−^	-
**7**	Luteolin-7,4′-di-*O*-rutinoside (dracopalmaside)	14.87	253, 265, 345	905 [M − H]^−^, 447 [(M − Rha − Glc) − H]^−^, 285 [(M – 2 × Rha – 2 × Glc) − H]^−^	ii [9]
**8**	Luteolin-7-*O*-rutinoside-4′-*O*-glucoside (cynarotriside)	15.02	253, 265, 345	755 [M − H]^−^, 609 [(M − Rha) − H]^−^, 447 [(M − Rha − Glc) − H]^−^, 285 [(M − Rha – 2 × Glc) − H]^−^	ii [9]
**9**	Eriodictyol-*O*-malonyl-hexoside (isomer)	15.41	283	535 [M − H]^−^, 449 [(M − malonyl) − H]^−^, 287 [(M − malonyl − Hex) − H]^−^	-
**10**	Eriodictyol-7-*O*-rutinoside (eriocitrin)	16.21	284	595 [M − H]^−^, 449 [(M − Rha) − H]^−^, 287 [(M − Rha − Glc) − H]^−^	i
**11**	Luteolin-7-*O*-rutinoside (scolymoside)	17.02	252, 262, 345	593 [M − H]^−^, 447 [(M − Rha) − H]^−^, 285 [(M − Rha − Glc) − H]^−^	i
**12**	Eriodictyol-7-*O*-glucoside	17.58	283	899 [2M − H]−, 449 [M − H]^−^, 287 [(M − Glc) − H]^−^	i
**13**	Luteolin-7-*O*-glucoside (cynaroside)	19.48	254, 267, 348	895 [2M − H]−, 447 [M − H]^−^, 285 [(M − Glc) − H]^−^	i
**14**	Luteolin-4′-*O*-glucoside	20.64	260, 335	447 [M − H]^−^, 285 [(M − Glc) − H]^−^	i
**15**	Apigenin-7-*O*-rutinoside (isorhoifolin)	22.51	266, 334	577 [M − H]^−^, 431 [(M − Rha) − H]^−^, 269 [(M − Rha − Glc) − H]^−^	i
**16**	Naringenin-7-*O*-glucoside (prunin)	23.89	283	433 [M − H]^−^, 271 [(M − Glc) − H]^−^	i
**17**	Apigenin-7-*O*-glucoside (cosmosiin)	24.47	267, 336	863 [2M − H]−, 431 [M − H]^−^, 269 [(M − Glc) − H]^−^	i
**18**	Apigenin-*O*-hexoside	25.31	265, 334	431 [M − H]^−^, 269 [(M − Glc) − H]^−^	iii [51]
**19**	Rosmarinic acid	27.26	327	359 [M − H]^−^, 183	i
**20**	Luteolin-*O*-acetyl-hexoside	29.11	251, 263, 346	489 [M − H]^−^, 447 [(M − acetyl) − H]^−^, 285 [(M − acetyl − Hex) − H]^−^	iii [52]
**21**	Eriodictyol	35.03	283	287 [M − H]^−^	i
**22**	Acacetin-7-*O*-rutinoside (linarin)	36.14	267, 330	591 [M − H]^−^, 445 [(M − Rha) − H]^−^, 283 [(M − Rha − Glc) − H]^−^	i
**23**	Acacetin-7-*O*-glucoside (tilianin)	36.72	266, 330	445 [M − H]^−^, 283 [(M − Glc) − H]^−^	i
**24**	Luteolin	37.72	253, 266, 347	285 [M − H]^−^	i
**25**	Acacetin-*O*-acetyl-hexoside	38.10	266, 331	487 [M − H]^−^, 445 [(M − acetyl) − H]^−^, 283 [(M − acetyl − Hex) − H]^−^	iii [52]
**26**	Naringenin	38.32	283	271 [M − H]^−^	i
**27**	Apigenin	39.75	267, 336	269 [M − H]^−^	i
**28**	Chrysoeriol	40.21	266, 347	299 [M − H]^−^	i
**29**	Acacetin	42.34	267, 330	283 [M − H]^−^	i
**30**	Isothymusin	43.06	302, 330	329 [M − H]^−^	ii [46]
**31**	Salvigenin	45.81	273, 330	327 [M − H]^−^	i
**32**	Genkwanin	46.04	267, 335	283 [M − H]^−^	i

^a^ Reference compound used: i, commercial sample; ii, isolated compound; iii, literature data.

**Table 5:** Content of phenolic compounds (mg/g DW ± SD) in *D. palmatum* herb under different temperatures of cultivation. ^1^

Compound (No of Compounds)	Temperature (°C)	
	20	1
Simple phenols		
*O*-Malonyl-arbutin (**1**)	0.39 ± 0.01 ^a^	1.24 ± 0.02 ^a^
Arbutin (**2**)	0.17 ± 0.00	0.22 ± 0.00
Subtotal	0.56	1.46
Phenylpropanoids		
5-*O*-Caffeoylquinic acid (**3**)	0.09 ± 0.00	0.05 ± 0.00
3-*O*-Caffeoylquinic acid (**4**)	0.11 ± 0.00	0.09 ± 0.00
Caffeic acid (**5**)	0.61 ± 0.02	0.84 ± 0.02
Rosmarinic acid (**19**)	1.26 ± 0.03	1.68 ± 0.04
Subtotal	2.07	2.66
Flavone glycosides. Apigenin derivatives		
Apigenin-7-*O*-rutinoside (isorhoifolin, **15**)	1.11 ± 0.03	0.56 ± 0.02
Apigenin-7-*O*-glucoside (cosmosiin, **17**)	0.53 ± 0.01	6.54 ± 0.17
Apigenin-*O*-hexoside (**18**)	0.47 ± 0.01 ^b^	8.34 ± 0.18 ^b^
Subtotal	2.11	15.44
Flavone glycosides. Acacetin derivatives		
Acacetin-7-*O*-rutinoside (linarin, **22**)	0.04 ± 0.00	0.06 ± 0.00
Acacetin-7-*O*-glucoside (tilianin, **23**)	0.52 ± 0.01	1.27 ± 0.04
Acacetin-*O*-acetyl-hexoside (**25**)	ND	0.08 ± 0.00 ^c^
Subtotal	0.56	1.41
Flavone glycosides. Luteolin derivatives		
Luteolin-7,4′-di-*O*-rutinoside (dracopalmaside, **7**)	0.14 ± 0.00	0.52 ± 0.01
Luteolin-7-*O*-rutinoside-4′-*O*-glucoside (cynarotriside, **8**)	0.82 ± 0.02	1.75 ± 0.04
Luteolin-7-*O*-rutinoside (scolymoside, **11**)	2.27 ± 0.07	2.54 ± 0.07
Luteolin-7-*O*-glucoside (cynaroside, **13**)	2.56 ± 0.07	29.56 ± 0.78
Luteolin-4′-*O*-glucoside (**14**)	0.67 ± 0.02	9.57 ± 0.19
Luteolin-*O*-acetyl-hexoside (**20**)	ND	0.92 ± 0.02 ^d^
Subtotal	6.46	44.86
Flavanone glycosides. Eriodictyol derivatives		
Eriodictyol-*O*-malonyl-hexoside (sum of **6** and **9**)	ND	1.84 ± 0.04 ^e^
Eriodictyol-7-*O*-rutinoside (eriocitrin, **10**)	0.21 ± 0.00	1.35 ± 0.03
Eriodictyol-7-*O*-glucoside (**12**)	1.77 ± 0.03	15.82 ± 0.33
Subtotal	1.98	19.01
Flavanone glycosides. Naringenin derivatives		
Naringenin-7-*O*-glucoside (pruning, **16**)	1.02 ± 0.02	1.64 ± 0.03
Subtotal	1.02	1.64
Flavone aglycones		
Luteolin (**24**)	1.19 ± 0.03	12.94 ± 0.30
Apigenin (**27**)	0.46 ± 0.01	1.03 ± 0.03
Chrysoeriol (**28**)	0.09 ± 0.00	0.14 ± 0.00
Acacetin (**29**)	ND	0.18 ± 0.00
Salvigenin (**31**)	ND	0.09 ± 0.00
Isothymusin (**30**)	ND	0.12 ± 0.00
Genkwanin (**32**)	ND	0.10 ± 0.00
Subtotal	1.74	14.60
Flavanone aglycones		
Eriodictyol (**21**)	0.24 ± 0.00	0.69 ± 0.02
Naringenin (**26**)	ND	0.54 ± 0.01
Subtotal	0.24	1.23
Total flavone glycosides	9.13	61.71
Total flavanone glycosides	3.00	20.65
Total flavonoids glycosides	12.13	82.36
Total flavonoids aglycones	1.98	15.83
Total flavonoids	14.11	98.19
Total phenolic compounds	16.74	102.31

^1^ Averages ± standard deviation were obtained from three different experiments. ^a^ Expressed as arbutin equivalents; ^b^ expressed as apigenin-7-*O*-glucoside equivalents; ^c^ expressed as acacetin-7-*O*-glycoside equivalents; ^d^ expressed as luteolin-7-*O*-glucoside equivalents; ^e^ expressed as eryodictyol-7-*O*-glucoside equivalents; ND—not detected.

**Table 6:** HPLC parameters, electrospray ionization mass spectrometry (ESI-MS) data and the content of simple sugars (mg/g DW ± SD) in *D. palmatum* herb under different temperatures of cultivation.

Compound	t_R_ (min)	ESI-MS (*m*/*z)*	Content (mg/g) ^1^	
			Temperature (°C)	
			20	1
Glucose	2.38	179 [M − H]^−^	16.86 ± 0.32	26.39 ± 0.52
Sucrose	2.73	341 [M − H]^−^	35.54 ± 0.78	169.21 ± 3.72
Stachyose	2.89	665 [M − H]^−^	1.72 ± 0.03	38.95 ± 0.82
Raffinose	3.04	503 [M − H]^−^	0.87 ± 0.02	9.36 ± 0.18
Total content			54.99	243.91

^1^ Averages ± standard deviations were obtained from three different experiments.

**Table 7:** Yield of raw water soluble polysaccharide (RWSP) of *D. palmatum* herb under different temperatures of cultivation, their general characteristics and monosaccharide compositions.

Parameter	Temperature (°C)	
	20	1
RWSP yield (%) ^a^	2.29 ± 0.04 ^c^	9.86 ± 0.20 ^c^
RWSP general characteristics		
Protein content, % ^b^	2.61 ± 0.07 ^c^	2.75 ± 0.09 ^c^
Uronic acids, % ^b^	43.57 ± 1.01 ^c^	46.16 ± 1.14 ^c^
Reaction with I_2_ (starch)	positive	positive
Reaction with resorcinol (inulin)	negative	negative
Reaction with Yariv’s reagent (AGP-complexes)	positive	positive
Reaction with Fehling’s reagent (mannans)	negative	negative
RWSP monosaccharide composition (mol %)		
Ara	10.1	10.2
Gal	26.1	27.7
Glc	14.4	10.2
Fuc	0.1	0.1
Man	4.3	4.0
Rha	1.9	1.7
Rib	Tr. ^d^	Tr. ^d^
Xyl	Tr. ^d^	Tr. ^d^
GalA	41.2	44.6
GlcA	1.8	1.4

^a^ Percentage of dry plant weight ± SD; ^b^ Percentage of dry RWSP weight ± SD; ^c^ Averages ± standard deviations were obtained from three different experiments; ^d^ Tr., traces (<0.1 mol %).

**Table 8:** Malondialdehyde (MDA) content, superoxide dismutase (SOD) and catalase activities of *D. palmatum* leaves and antioxidant activity of *D. palmatum* extracts obtained from the herb grown under different temperatures of cultivation and luteolin-7-*O*-glucoside as a reference compound ^a,b^.

Parameter	Luteolin-7-*O*-Glucoside	Temperature (°C)	
		20	1
MDA content (nM/g) FW	-	92.74 ± 7.41	197.02 ± 15.76
SOD activity (U/g·min) FW	-	57.90 ± 5.21	264.32 ± 12.35
Catalase activity (U/g·min) FW	-	0.93 ± 0.06	1.53 ± 0.12
Total antioxidant capacity (mg-eq). luteolin-7-*O*-glucoside/g	1000 ^iii^	280.98 ± 8.99 ^i^	682.26 ± 21.15 ^ii,iii^
DPPH^•^-radical scavenging activity, IC_50_ (µg/mL)	16.97 ± 0.34 ^iv,v^	33.28 ± 0.73 ^vi^	11.40 ± 0.24 ^iv^
ABTS^•+^-radical scavenging activity, IC_50_ (µg/mL)	9.86 ± 0.19 ^vii,viii^	14.62 ± 0.31 ^viii^	5.69 ± 0.11 ^vii^
O_2_^•−^-radical scavenging activity, IC_50_ (µg/mL)	14.92 ± 0.43 ^ix,x^	18.36 ± 0.56 ^x^	9.21 ± 0.21 ^ix^
Br^•^-radical scavenging activity (mg-eq). luteolin-7-*O*-glucoside/g	1000 ^xii^	150.19 ± 1.95 ^xi^	799.63 ± 11.19 ^xii^
NO inactivating activity, IC_50_ (µg/mL)	>100	37.92 ± 1.59	21.37 ± 0.85 ^xiii^
H_2_O_2_ inactivating activity (mM/g)	0.53 ± 0.02 ^xiv^	1.56 ± 0.04	2.75 ± 0.06 ^xv^
Fe^2+^-chelating activity (µM) Fe^2+^/g	106.12 ± 3.18 ^xvi^	142.84 ± 4.42 ^xvi,xvii^	206.11 ± 4.78 ^xviii^

^a^ Averages ± standard deviations (SD) were obtained from five different experiments; ^b^ Values with different roman letters (^i^–^xviii^) indicate statistically significant differences among groups at *p* < 0.05 by one-way analysis of variance.
